# Human Chr18 transcriptome dataset combined from the Illumina HiSeq, ONT MinION, and qPCR data

**DOI:** 10.1016/j.dib.2021.107130

**Published:** 2021-05-12

**Authors:** George Krasnov, Timur Shkrigunov, Sergey Radko, Konstantin Ptitsyn, Valeriya Shapovalova, Olga Timoshenko, Svetlana Khmeleva, Leonid Kurbatov, Yana Kiseleva, Ekaterina Ilgisonis, Olga Kiseleva, Igor Vakhrushev, Anastasia Tsvetkova, Ivan Buromski, Sergey Markin, Alexander Archakov, Andrey Lisitsa, Elena Ponomarenko

**Affiliations:** a Institute of Biomedical Chemistry, Moscow, Russia; b Engelhardt Institute of Molecular Biology, Russian Academy of Sciences, Moscow, Russia; c Mechnikov Research Institute of Vaccines and Sera, Moscow, Russia; d Centre for Strategic Planning and Management of Biomedical Health Risks, Federal Medical Biological Agency, Moscow, Russia; e Russian Scientific Center of Roentgen Radiology, Moscow, Russia; f Department of Forensic Medicine, Faculty of General Medicine, Pirogov Russian National Research Medical University, Moscow, Russia

**Keywords:** Human proteome project, Illumina HiSeq, Oxford nanopore Technologies, Transcriptome, HepG2, Liver tissue

## Abstract

The chromosome-centric dataset was created by applying several technologies of transcriptome profiling. The described dataset is available at NCBI repository (BioProject ID PRJNA635536). The dataset referred to the same type of tissue, cell lines, transcriptome sequencing technologies, and was accomplished in a period of 8 years (the first data were obtained in 2013 while the last ones — in 2020). The high-throughput sequencing technologies were employed along with the quantitative PCR (qPCR) approach, for data generation using the gene expression level assessment. qPCR was performed for a limited group of genes, encoded on human chromosome 18, for the Russian part of the Chromosome-Centric Human Proteome Project. The data of high-throughput sequencing are provided as Excel spreadsheets, where the data on FPKM and TMP values were evaluated for the whole transcriptome with both Illumina HiSeq and Oxford Nanopore Technologies MinION sequencing.

**Specifications Table**

**Table:** 

Subject	Biological sciences — Biochemistry
Specific subject area	Chromosome-Centric Human Proteome Project
Type of data	Table Figure
How data were acquired	Quantitative PCR (RT-PCR or ddPCR); high throughput RNA sequencing by Illumina HiSeq 2500 system and ONT MinION nanopore sequencer
Data format	Raw (FastQ) Analyzed (FPKM, TPM) Filtered (Chr18 genes)
Parameters for data collection	RNA preparations from three human hepatocellular carcinoma samples and one sample of cultured HepG2 cells were used to analyze: (1) total RNA for profiling the Chr18 transcriptome by quantitative PCR; (2) amplified polyA^+^ RNA for the whole transcriptome sequencing with Illumina HiSeq; (3) extracted polyA^+^ RNA for the direct sequencing of whole transcriptome with ONT MinION.
Description of data collection	The samples collected were immediately placed in RNAlater RNA stabilization solution (Thermo Fisher Scientific) and stored at −20°C. Total RNA extraction was performed using RNeasy Mini Kit (Qiagen). The rRNA-depleted RNA was obtained with Ribo-Zero rRNA depletion kit (Illumina). The polyA^+^ RNA was isolated with Dynabeads mRNA Purification Kit (Thermo Fisher Scientific). The sequencing libraries construction and sequencing were carried out according to manufacturers’ protocols. The PCR analysis was conducted in duplicates, Illumina HiSeq sequencing – with 2 replicates, ONT MinION sequencing – with a single run.
Data source location	Institute of Biomedical Chemistry, Moscow, Russia 55.732560, 37.567401
Data accessibility	**2013 Data** With the article http://dx.doi.org/10.1021/pr400883x **2020 Data** Repository name: NCBI Database: BioProject Accession: PRJNA635536 Direct URL to data: https://www.ncbi.nlm.nih.gov/sra?linkname=bioproject_sra_all&from_uid=635536 **Table S1** Repository name: Mendeley Data Direct URL to data: https://data.mendeley.com/datasets/nwkr6z9g4z/1
Related research article	K.A. Deinichenko, G.S. Krasnov, S.P. Radko, K.G. Ptitsyn, V. V Shapovalova, O.S. Timoshenko, S.A. Khmeleva, L.K. Kurbatov, Y.Y. Kiseleva, E. V Ilgisonis, M.A. Pyatnitskiy, E. V Poverennaya, O.I. Kiseleva, I. V Vakhrushev, A. V Tsvetkova, I. V Buromski, S.S. Markin, V.G. Zgoda, A.I. Archakov, A. V Lisitsa, E.A. Ponomarenko, Human Chr18: "Stakhanovite" Genes, Missing and uPE1 Proteins in Liver Tissue and HepG2 Cells, Biomedical Chemistry: Research and Methods (2021) 4, e00144. http://www.bmc-rm.org/index.php/bmcrm/article/view/144/360

## Value of the Data

• Data is necessary for versatile exploration of cross-correlation between transcriptome analytical platforms, including the quantitative PCR (qPCR) for targeted analysis of gene expression, combined with the gene expression analysis by the short read (Illumina HiSeq) and long read (ONT MinION) RNA-seq technologies.
• The Chromosome-Centric Human Proteome Project community could use the data to decipher the tissues-specific missing proteins, i.e., transcriptome sequence for which a protein product was not detected yet.
• The data could be beneficial for the analysis of splice-variants of genes, which are involved in the physiological and pathophysiological pathways of liver drug-metabolizing system.

## 1 Data Description

**Fig. 1:**
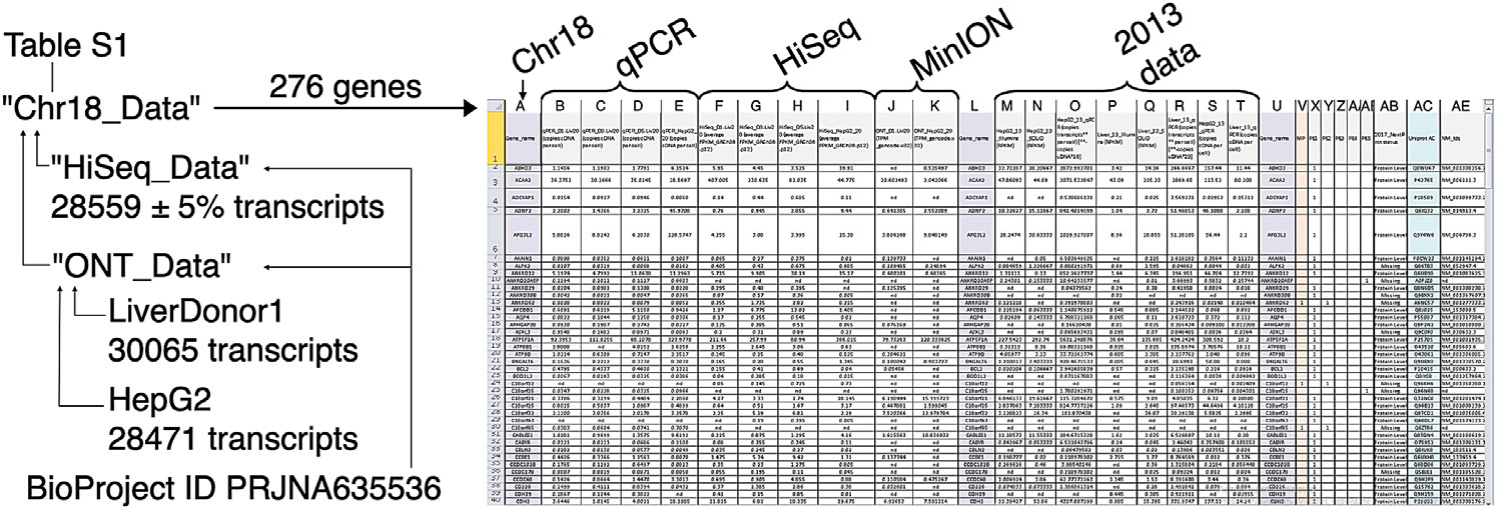
Dataset origin and structure. Quantitative data for samples of liver tissue and HepG2 cells from several datasets, obtained in 2013 and 2020 with SOLiD, Illumina GII/HiSeq, qPCR and Oxford Nanopore (MinION), and mapped to the Chr18-encoded proteins.

We present our data as the supplementary Table S1. In this table, we have combined the data for the genes of chromosome 18 (see Fig. 1) derived with RNA-Seq and qPCR methods. The RNA-seq quantitative data for the whole transcriptome are also provided in the same file. The RNA-seq was performed for four specimens, the same as used for the qPCR analysis, which were post-mortal samples of liver tissue from three donors and the sample of HepG2 cells.

The presented data contains three basic spreadsheets. In “HiSeq_Data” worksheet, the data on whole transcriptome sequencing by the Illumina HiSeq were provided. “ONT_Data” page presents the data which were provided by sequencing samples of HepG2 cells and liver tissue from donor 1 with the Oxford Nanopore Technologies MinION sequencer. In the “Chr18_data" page of Table S1 the data on chromosome 18 genes are provided.

The following information is presented at the “Chr18_Data” worksheet: the first section is the data which were obtained recently, in 2020 (see Fig. 1). The first four columns, from B to E, are devoted to the qPCR analysis, where the data on gene expression are presented as the number of copies of cDNA per cell (average of duplicate measurements). The next four columns, from F to I, contain the information received via HiSeq sequencing, where gene expression is given in FPKM (average of two technical replicates). To obtain FPKM values, the GRCh38.p12 genome assembly was used. The J column describes Oxford Nanopore data for liver tissue of donor 1 (Donor1.Liver) and the column K describes those for HepG2 cells. Here, the data are expressed in TPM values (transcript per million) and these values were calculated using the GRCh38.p13 transcript assembly.

The next section (columns M to T) contains information, which was obtained in 2013, at the initial stage of the project. The Illumina Gallx and SOLiD sequencing, as well as qPCR analysis, were performed on HepG2 cells and a sample obtained by pooling post-mortal liver tissue specimens from three donors [1,2].

Finally, the last section of Table S1 contains the list of some genes whose putative protein products are defined with the protein evidence level by the UniProt database or as “missing proteins”. The UniProt accession numbers and NM identifiers for the gene products are also given.

**Fig. 2:**
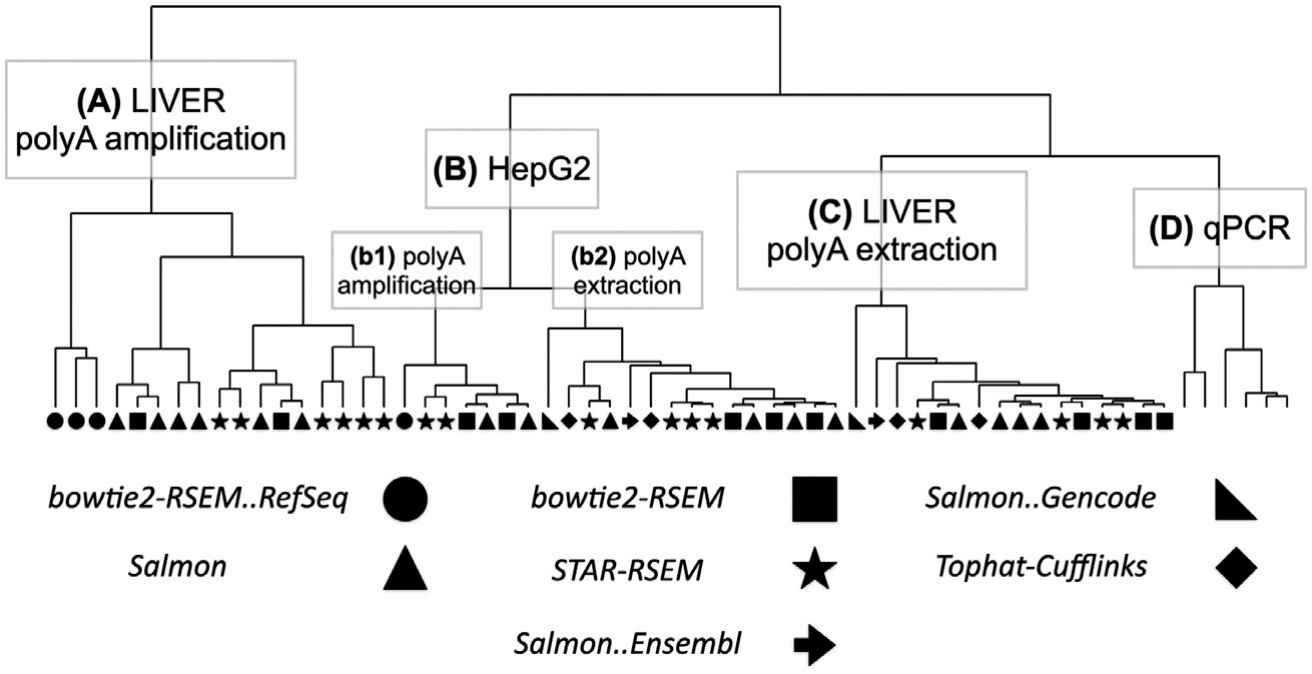
Relationships between clusters within dataset. Each leaf in the dendrogram corresponds to the certain source of the data about the expression level of Chromosome 18 genes, measured with different methods and processed with different bioinformatics pipelines (bowtie2-RSEM..RefSeq, Salmon, bowtie2-RSEM, STAR-RSEM, Salmon..Gencode, Tophat-Cufflinks, Salmon..Ensembl).

Clustering was carried out using Ward's D2 method based on the values of Spearman rank correlation coefficients (Fig. 2). The dendrogram in the figure illustrates four main clusters: A, B, C, D. Note that clusters A and C both relate to the liver specimens, but are different in methods of mRNA enrichment. The similar situation is observed for cluster B, which can be split into two subclusters b1 and b2, corresponding to the polyA amplification and polyA extraction approaches for the sample preparation. The least number of elements is observed for the cluster D which combines data acquired by qPCR for 6 biological specimens tested. The cluster B is composed by the data for HepG2 cell samples and contains the largest number of elements – 24. The correlational relations between elements of each cluster are provided in Table 1.

**Table 1:** Properties of the dataset. Headers of rows and columns denote clusters formed as a result of the correlation analysis between Chr18 transcriptome profiles derived by various methods (qPCR or RNA-seq) for different types of specimen (human liver or cultured HepG2 cells).

Main clusters	LIVER polyA amplification (n = 17)	HepG2 (n = 22)	LIVER polyA extraction (n = 16)	qPCR (n = 6)
**LIVER** **polyA amplification** **(n = 17)**	*r*_s_=0.79 ± 0.10 *r*_p_=0.95 ± 0.04	*r*_s_=0.67 ± 0.10	*r*_s_=0.73 ± 0.10	*r*_s_=0.55 ± 0.08

**HepG2** **(n = 22)**	*r*_p_=0.63 ± 0.09	*r*_s_=0.90 ± 0.06 *r*_p_=0.86 ± 0.10	*r*_s_=0.77 ± 0.07	*r*_s_=0.66 ± 0.09

**LIVER** **polyA extraction** **(n = 16)**	*r*_p_=0.94 ± 0.04	*r*_p_=0.65 ± 0.09	*r*_s_=0.93 ± 0.07 *r*_p_=0.97 ± 0.02	*r*_s_=0.70 ± 0.07

**qPCR** **(n = 6)**	*r*_p_=0.63 ± 0.22	*r*_p_=0.55 ± 0.10	*r*_p_=0.67 ± 0.22	*r*_s_=0.83 ± 0.09 *r*_p_=0.71 ± 0.17

*r*_p_*— Pearson correlation coefficient; r*_s_*— Spearman rank correlation coefficient*

± — standard deviation

To present our data in the generalized tabular format (Table 1) we averaged the correlation coefficients within the main clusters shown in Fig. 2: LIVER polyA amplification, HepG2, LIVER polyA extraction and qPCR. The bottom-left part of the table presents the means and corresponding standard deviations for values of Pearson correlation coefficients within each cluster. The top-right part — the same representation for values of Spearman rank correlation coefficients. The numbers of leaves for each cluster from Fig. 2 are shown as “n” in the Table 1.

## 2 Experimental Design, Materials and Methods

### 2.1 Specimens

Human liver specimens were collected at autopsy from three male donors aged 65, 38, and 54 years, two of whom died due to acute cardiovascular insufficiency and one – due to the trauma. The samples were immediately placed into the RNAlater RNA Stabilization Solution (Thermo Fisher Scientific, USA) and stored at -20°C until further use. Prior to analysis, the RNA integrity numbers (RINs) were measured and found to be in the range of 7.5–9.0.

HepG2 cells (ATCC HB-8065) were grown in culture medium (DMEM/F12 supplemented with 10% fetal bovine serum (FBS) and 100 units/ml penicillin/streptomycin (all from Gibco, USA)) in a humidified CO_2_-incubator under standard conditions (5% CO_2_, 37 °C). The medium was exchanged every 2 days. After reaching approximately 80% confluence, the cells were detached with 0.25% Trypsin-EDTA solution (PanEco, Russia), washed 3 times with PBS, and counted with an EVE automated cell counter (NanoEntek, South Korea). Afterwards, cells were pelleted by centrifugation and kept in liquid nitrogen until further use.

### 2.2 qPCR data

The qPCR data are presented as the number of biomolecule copies per cell. In order to derive such units, we quantified the concentration of total RNA and then used it to normalize the output of PCR measurements. For transcriptome profiling with qPCR, total RNA was isolated from liver tissue samples and HepG2 cells using the RNeasy Mini Kit (Qiagen, Germany) according to the manufacturer's protocol. The on-column DNase digestion step was performed using the RNase-Free DNase Set (Qiagen, Germany). The isolated total RNA was quantified using a Qubit 4 fluorometer and the Qubit RNA HS Assay Kit (Thermo Fisher Scientific, USA), and the RNA quality was assessed using a Bioanalyzer 2100 System (Agilent Technologies, USA). Synthesis of cDNA was carried out using the AffinityScript qPCR cDNA Synthesis Kit and random primers (Agilent Technologies, USA) according to the manufacturer's recommendations. The cDNA samples were stored at −20°C until further use. The amount of each transcript encoded on Chr18 was assessed by measuring the number of pertinent cDNA copies, using the set of primers and probes developed earlier [1], [2], [3]. All PCR measurements were made in duplicates and the average values were used as estimates. For real-time PCR, the quantification was carried out employing the ΔCT-method [4] and a group of reference transcripts whose absolute concentrations were determined as described previously [1,2]. The number of transcripts per nanogram of total RNA was brought to the copy numbers per cell, based on the amount of total RNA in hepatocytes and HepG2 cells, reported to equal 40 pg/cell [5].

### 2.3 Illumina HiSeq data

To generate the HiSeq part of the dataset, each specimen of the human liver tissue was split into two pieces which were analyzed independently. Total RNA was isolated using Extract RNA kit (Evrogen, Russia). RNA integrity was evaluated using both capillary electrophoresis by Bioanalyzer 2100 System (Agilent Technologies, USA) and agarose electrophoresis. RIN numbers varied from 7.3 to 9.1. Next, we synthesized full-length-enriched double stranded cDNA using Mint-2 kit (Evrogen, Russia). Briefly, oligo(dT) primers were annealed to poly(A) 3’-tails of RNA. Methodologically important to highlight that when Mint reverse transcriptase reaches the 5’-end of the mRNA, it adds several non-template nucleotides, primarily deoxycytidines, to the 3’-end of the newly synthesized first-strand cDNA. So, these properties of Mint transcriptase enabled annealing oligo(gG) primers (“PlugOligo”) to the 5’-tails and synthesis of the second cDNA strand. Next, we prepared sequencing-ready cDNA libraries using Qiaseq FX DNA Library Kit (Qiagen, USA) according to the manufacturer's protocol. Library quality control was carried out using Bioanalyzer 2100 System (Agilent Technologies, USA) in order to evaluate insert distribution. Clustering and sequencing were carried out using Illumina HiSeq 2500 system (2 lanes per 8 samples) according to the manufacturer's protocols (Denature and Dilute Libraries Guide; Sequencing in Rapid Run Mode). For each replicate, we derived from 32 to 59 million reads.

The derived fastq files were analyzed by FastQC and then processed by trimmomatic. Then, we proceed several ways. First, we mapped reads to the genome GRCh38.p12 assembly using STAR 2.7 with 1) provided GTF annotation; 2) enabled search for novel splice junctions (only canonical splice sites); 3) two output BAMs: in genomic coordinates and in transcript coordinates. The last one was used to quantify genes and transcripts expression by RSEM 1.3, either in terms of FPKM (fragments per kilobase per million) and TPM (transcripts per million).

Second, we mapped reads directly to reference transcripts and quantified expression. For this purpose, we reconstructed GRCh38.p12 transcripts sequences and created bowtie2 index with RSEM (rsem-prepare-reference), then mapped reads to the transcripts using bowtie2 and finally quantified expression using RSEM (rsem-calculate-expression; both TPM and FPKM). Additionally, we also used Salmon to evaluate gene/transcripts expression by pseudo-mapping reads to the GRCh38.p12 transcripts.

### 2.4 ONT MinION data

The nanopore sequencing platform developed by the Oxford Nanopore Technology (ONT, United Kingdom) was used to characterize the biosamples from the liver tissue of donor 1 and HepG2 cell line. The extraction of mRNA from the total RNA preparations was conducted using the Dynabeads mRNA Purification Kit (Thermo Fisher Scientific, USA) following the manufacturer's recommendations. The mRNA preparations were immediately frozen and stored at −80°C until nanopore sequencing. Nanopore sequencing was carried out using the MinION sequencer (ONT, UK) with FLO-MIN106 flow cells and R9.4 chemistry and the Direct RNA sequencing kit (SQK-RNA002, ONT, UK). The sequencing libraries were prepared strictly following the manufacturer's protocol with a single exception: 750 ng of mRNA (poly+ RNA) was used as the input in both samples from the human liver and HepG2 cells instead of the recommended 500 ng. The SuperScript III Reverse Transcriptase (Thermo Fisher Scientific, USA) was used for reverse transcription and NEBNext Quick Ligation Module (New England Laboratories, UK) was used for end repair and ligation. The Agencourt RNAClean XP magnetic beads (Beckman Coulter, USA) were employed for nucleic acid purification. The mRNA from HepG2 was sequenced in a 72-h single run. The output was 0.75 Gb sequenced transcripts (0.766 million reads) with a median length of 1.56 kb. The mRNA from the tissue liver of donor 1 was sequenced for 26 h. The flow cell was regenerated using the Flow Cell Wash Kit (ONT, UK), strictly following the manufacturer's guidance. Next, the newly prepared sequencing library from the liver mRNA of donor 1 was loaded on the flow cell and a 48-h sequencing run was initiated. The overall output was 1.44 million reads with a median length of 1.37 kb.

The fast5 files produced by MinION were uploaded onto the Amazon Web Services ElasticCloud2 and processed using the GPU-powered (NVidia Tesla V100) virtual instance p3.2xlarge (8 × 2.7 GHz vCPUs, 1 GPU) by the guppy basecaller 3.6.1 [6]. Mapping the reads onto the GRCh38.p13 transcripts assembly was performed by minimap2 2.17 [7]. Salmon 1.1.0 tool was used to quantify the transcripts [8]. For both Illumina HiSeq and ONT MinION data the gene expression levels were also derived by summarizing the values of the expression levels of transcripts corresponding to the same gene.

## Ethics Statement

Samples of human liver tissue were collected at autopsy from 3 male donors (designated as donors 1, 3, and 5) aged 65, 38, and 54 years with the approval of the Ethical Committee of the N. I. Pirogov Russian National Research Medical University (Protocol #3; March 15, 2018) with the informed consent from donor's representatives.

## CRediT Author Statement

**George Krasnov:** RNA-seq bioinformatics and manuscript drafting; **Sergey Radko:** wet-lab experiments coordination and manuscript preparation; **Konstantin Ptitsyn:** ONT experiments; **Valeriya Shapovalova:** ONT-bioinformatics; **Olga Timoshenko and Leonid Kurbatov:** samples preparation; **Svetlana Khmeleva:** PCR experiments; **Yana Kiseleva:** ddPCR experiments; **Ekaterina Ilgisonis:** data analysis and visualization; **Olga Kiseleva:** data processing; **Igor Vakhrushev and Anastasia Tsvetkova:** HepG2 cells management; **Ivan Buromski and Sergey Markin:** liver biospecimens collection; **Alexander Archakov:** author of the idea; **Andrey Lisitsa and Timur Shkrigunov:** ONT-bioinformatics, technical writing; **Elena Ponomarenko:** general coordination, workflows, and data integration.

## Declaration of Competing Interest

The authors declare that they have no known competing financial interests or personal relationships which have or could be perceived to have influenced the work reported in this article.
